# Low risk of recurrence following artesunate–Sulphadoxine–pyrimethamine plus primaquine for uncomplicated *Plasmodium falciparum* and *Plasmodium vivax* infections in the Republic of the Sudan

**DOI:** 10.1186/s12936-018-2266-9

**Published:** 2018-03-16

**Authors:** Muzamil Mahdi Abdel Hamid, Kamala Thriemer, Maha E. Elobied, Nouh S. Mahgoub, Salah A. Boshara, Hassan M. H. Elsafi, Suhaib A. Gumaa, Tassneem Hamid, Hanadi Abdelbagi, Hamid M. Basheir, Jutta Marfurt, Ingrid Chen, Roly Gosling, Ric N. Price, Benedikt Ley

**Affiliations:** 1 Department of Parasitology and Medical Entomology, Institute of Endemic Diseases, University of Khartoum, Khartoum, Republic of the Sudan; 2 Global and Tropical Health Division, Menzies School of Health Research and Charles Darwin University, Casuarina, PO Box 41096, Darwin, NT 0811 Australia; 3 Global Health Group, University of California San Francisco, San Francisco, CA USA; 4 Centre for Tropical Medicine and Global Health, Nuffield Department of Clinical Medicine, University of Oxford, Oxford, UK

## Abstract

**Background:**

First-line schizontocidal treatment for uncomplicated malaria in the Republic of the Sudan is artesunate (total dose 12 mg/kg) plus Sulphadoxine/pyrimethamine (25/1.25 mg/kg) (AS/SP). Patients with *Plasmodium vivax* are also treated with 14 days primaquine (total dose 3.5 mg/kg) (PQ). The aim of this study was to assess the efficacy of the national policy.

**Methods:**

Patients above 1 year, with microscopy-confirmed, *Plasmodium falciparum* and/or *P. vivax* malaria were treated with AS/SP. Patients with *P. falciparum* were randomized to no primaquine (Pf-noPQ) or a single 0.25 mg/kg dose of PQ (Pf-PQ1). Patients with *P. vivax* received 14 days unsupervised 3.5 mg/kg PQ (Pv-PQ14) on day 2 or at the end of follow up (Pv-noPQ). Primary endpoint was the risk of recurrent parasitaemia at day 42. G6PD activity was measured by spectrophotometry and the Accessbio Biosensor™.

**Results:**

231 patients with *P. falciparum* (74.8%), 77 (24.9%) with *P. vivax* and 1 (0.3%) patient with mixed infection were enrolled. The PCR corrected cumulative risk of recurrent parasitaemia on day 42 was 3.8% (95% CI 1.2–11.2%) in the Pf-noPQ arm compared to 0.9% (95% CI 0.1–6.0%) in the Pf-PQ1 arm; (HR = 0.25 [95% CI 0.03–2.38], p = 0.189). The corresponding risks of recurrence were 13.4% (95% CI 5.2–31.9%) in the Pv-noPQ arm and 5.3% (95% CI 1.3–19.4%) in the Pv-PQ14 arm (HR 0.36 [95% CI 0.1–2.0], p = 0.212). Two (0.9%) patients had G6PD enzyme activity below 10%, 19 (8.9%) patients below 60% of the adjusted male median. Correlation between spectrophotometry and Biosensor™ was low (r_s_ = 0.330, p < 0.001).

**Conclusion:**

AS/SP remains effective for the treatment of *P. falciparum* and *P. vivax*. The addition of PQ reduced the risk of recurrent *P. falciparum* and *P. vivax* by day 42, although this did not reach statistical significance. The version of the Biosensor™ assessed is not suitable for routine use.

*Trial registration* https://clinicaltrials.gov/ct2/show/NCT02592408

**Electronic supplementary material:**

The online version of this article (10.1186/s12936-018-2266-9) contains supplementary material, which is available to authorized users.

## Background

There has been a decline in the incidence of malaria in many sub-Saharan countries in the last decade [1–3], which has been attributed to the introduction of artemisinin-based combination therapy (ACT), the scaling up of the distribution of insecticide-impregnated bed nets (ITNs) and indoor residual spraying (IRS) [4]. There has also been an unprecedented increase in the funding of malaria control activities including intensive case management.

In Sudan, the number of malaria cases has declined, although the exact reduction in the burden of disease remains unclear. Information from local studies and reports suggest that in 2002 there were an estimated 9 million clinical cases of malaria and 44,000 deaths [5]. The WHO estimates are lower but highlight a substantial decline between 2000 and 2015, cases falling from 4 million cases in 2000–586,827 cases in 2015, with a total of 868 deaths [4]. However these successes have been associated with an increase in the proportion of infections due to *Plasmodium vivax* [6]. This rise likely reflects malaria control activities mostly targeting *Plasmodium falciparum* [7], and possibly an increased importation of vivax cases from neighbouring countries [6, 8, 9].

Since 2004, the first-line schizontocidal treatment for uncomplicated malaria in the Republic of Sudan has been artesunate plus Sulphadoxine–pyrimethamine (AS/SP) for both *P. falciparum* and *P. vivax* infections [10, 11] (Additional file 1, p. 12–34). In addition primaquine (PQ) is prescribed as a 14 day regimen for the radical cure of *P. vivax* hypnozoites following World Health Organization (WHO) guidelines (total dose 3.5, 0.25 mg/kg daily) [12, 13]; a single dose PQ is not currently recommended for patients with *P. falciparum* as gametocytocidal [14].

Whilst PQ is generally well tolerated, it can cause severe haemolysis in individuals with glucose 6 phosphate dehydrogenase deficiency (G6PDd) [15]. The WHO recommends that where feasible all patients should be tested for G6PD deficiency prior to prescribing primaquine based radical cure, however in view of the lack of available point of care tests to determine G6PDd status, this is not stipulated in the Sudanese anti-malarial treatment policy.

The aim of this study was to assess the efficacy and safety of AS/SP in patients with *P. falciparum* or *P. vivax* infection and the additional benefit of PQ either as a single dose for *P. falciparum* or 14-day regimen for *P. vivax*. The Biosensor™ (Accessbio, USA), a novel quantitative point of care G6PD diagnostic was also evaluated.

## Methods

### Study sites

The study was conducted at two sites in the Republic of Sudan: Gezira Slanj, a semi-urban area north of Khartoum (15°53′11.2″N 32°31′39.9″E), and New Halfa, in the east of the country (15°20′N 35°35′00″E). Malaria transmission in north Khartoum and eastern Sudan is seasonal with the main transmission occurring between September to February [16, 17], although malaria occurs throughout the year at both study sites. Key mosquito vectors in Sudan include *Anopheles arabiensis*, besides *Anopheles gambiae* s.l. and *Anopheles funestus* s.l. [11].

### Study population

Study participants were recruited from patients presenting to one of two health care facilities with febrile illness. Patients with peripheral *P. falciparum* and/or *P. vivax* parasitaemia detected by microscopy, aged at least 12 months, with history of fever in the last 24 h or axillary body temperature ≥ 37.5 °C were eligible for enrolment. Patients were excluded if they weighed less than 5 kg, were pregnant, had signs of severe malaria [18], were unable to tolerate oral medication, had a haemoglobin (Hb) concentration less than 8 g/dl, were on regular medication which could interfere with study treatment, or had known allergies to any of the study drugs. Whenever G6PD activity was determined by spectrophotometry participants with G6PD activities below 3.0 U/gHb were excluded from 14 day primaquine treatment [19].

### Study design and randomization

The study was designed as an open label, prospective randomized, controlled trial with 42 days of follow up. All patients were treated with AS/SP, allocation to PQ treatment was randomized according to the species of infection. Patients with *P. falciparum* mono-infection were randomized to receive AS/SP either alone (Pf-noPQ) or with a single dose of PQ (Pf-PQ1) administered on day 2. Patients with a *P. vivax* mono or mixed infection were randomized to receive AS/SP either alone (Pv-noPQ) or combined with 14 days of PQ (Pv-PQ14) starting on day 2. Patients in the Pv-noPQ arm received PQ according to national guidelines but delayed until after the end of 42 day follow up.

Treatment allocation was randomized in blocks of 20 and was provided in a sealed opaque envelope, which was only opened by a study nurse once the participant had met all the enrolment criteria and had provided written informed consent. Study staff assigned the patients sequentially according to the sealed envelopes. Blinding of clinical staff towards group allocation was not possible with the standard commercially sourced drugs; however, assignment was concealed from the microscopists responsible for determination of the primary endpoint.

### Treatment

Quality assured co-formulated blister packs of AS/SP (Shanghai-Sudan Pharmaceutical Ltd, China) were used for the study and patients were treated for 3 days according to national guidelines (target dose 4 mg/kg/day for AS and 25/1.25 mg/kg for SP) [13, 14]. On day 2, patients with *P. falciparum* infection were allocated to receive either no further treatment (Pf-noPQ) or a single dose of 0.25 mg/kg PQ (Pf-PQ1), manufactured by ipca Laboratories Ltd, Mumbai India.

Patients infected with *P. vivax* alone or mixed with *P. falciparum*, were allocated either to AS/SP and 14 days of unsupervised PQ (0.25 mg/kg/day, total dose 3.5 mg/kg) starting on day 2 (Pv-PQ14) or AS/SP alone (Pv-noPQ).

All doses of AS/SP and the single dose PQ in the Pf-PQ1 arm were supervised, however only the first dose of primaquine in the Pv-PQ14 arm was supervised. Study participants were observed for 30 min after administration and those vomiting their dose were treated again with the same dose and observed for a further 30 min.

Patients with recurrent uncomplicated malaria during the follow up period were treated with artemether–lumefantrine (AL) and patients with severe malaria were treated with quinine according to national guidelines [14].

### Clinical procedures and follow up

At enrolment demographic data, symptom details and history of anti-malarial medication were collected in a standardized questionnaire. A clinical examination was carried out and up to 5 ml venous blood taken for blood film examination, measurement of haemoglobin concentration (Hb), analysis of G6PD enzyme activity and parasite genotyping. Haemoglobin was measured at the bedside using at Hemocue 201 (Hemocue, Sweden) and G6PD activity measured with an experimental Biosensor™ (Accessbio, USA) following procedures as recommended by the manufacturer and described elsewhere [20, 21].

Patients were examined daily for the first 3 days and thereafter weekly on days 7, 14, 21, 28, 35 and 42. Patients with *P. vivax* were seen on day 16 rather than day 14, at which time a pill count was undertaken. Patients were encouraged to return to the clinic whenever they experienced signs and symptoms consistent with malaria. At each follow up visit, a full physical examination was undertaken, a symptom questionnaire completed, adverse events assessed and capillary blood collected for repeat blood film examination and Hb measurement. Whenever possible patients who did not return for scheduled follow up visits were contacted and encouraged to return.

### Laboratory procedures

#### Microscopy

Thick and thin films were stained with Giemsa and counted per 200 white blood cells (WBC) or 1000 red blood cells (RBC). Presence and density of gametocytes were also assessed per 200WBC. Slides for microscopy were subject to internal and external quality control checks. 20% of all slides were read twice by two local microscopists, and in case of discordant results reread by a third microscopist (internal QC). All slides collected on enrolment and at the time of recurrent parasitaemia were subject to external quality control by a WHO-certified expert microscopist of the Malaria Research Facility of the Papuan Health and Community Development Foundation (PHCDF) in Timika, Papua Province, Indonesia (external QC).

#### G6PD testing

Due to logistical reasons G6PD activity was only measured at one of the two sites. In New Halfa G6PD activity was measured directly upon sample collection using a handheld Biosensor (AccessBio CareStart, USA) and within 24 h by spectrophotometry (SPINREACT™, Spain) on a Mindray BA-88A (Minddray, China). G6PD normal and deficient controls (Ref: 100 252 0, SPINREACT, Spain), were run daily prior to sample measurement. Patients with *P. vivax* infection and G6PD activity below 3.0 U/gHb (based on spectrophotometry) were excluded from PQ treatment [19].

#### PCR testing

DNA was extracted from whole blood with a QIAamp DNA blood macro kit (Qiagen) following the manufacturer’s recommendations. Extracted DNA was eluted in 50 μl water and used for the genotyping of recurrent *P. falciparum* infections following WHO recommendations [22] by characterizing the length polymorphism of the *msp1*, *msp2*, and *glurp* genes in samples collected at day 0 and on the day recurrent parasitaemia was found.

Recrudescence was determined when, for each marker (*msp1*, *msp2*, and *glurp*), at least one identical-length polymorphism was found between samples collected on day 0 and on the day of recurrent infection. A new infection was defined when, for at least one marker, the length polymorphisms were different between the sample collected on day 0 and that collected on the day of recurrent infection. Samples were defined as indeterminate when no marker could be amplified.

### Study endpoints and definitions

The primary endpoint of the study was the rate of recurrence of peripheral asexual parasitaemia within 42 days of follow up. Secondary endpoints were the proportion of patients with parasitaemia or fever on days 1, 2 and 3 and gametocyte carriage following *P. falciparum* malaria. Response to treatment was defined according to WHO definitions [23] and treatment failures were categorized as early treatment failures (ETF), late parasitological (LPTF) and late clinical treatment failures (LCTF). Patients without asexual parasitaemia by day 42, and who did not previously meet any of the criteria of ETF, LPTF or LCTF were defined as adequate clinical and parasitological response (ACPR). Fever clearance time (FCT) in patients with a temperature ≥ 37.5 °C at enrolment was defined as the time from drug administration until body temperature (axillary) was below 37.5 °C.

### Statistical analysis

Assuming that AS/SP has a 15% risk of treatment failure at day 42 [24], and that this is unaffected by the addition of a single dose of primaquine, a total of 235 patients with *P. falciparum* infection determine the efficacy of AS/SP within ± 5% allowing for a loss to follow up rate of 10%. To achieve 80% power, 95% significance level to detect a decrease in *P. vivax* recurrence from 30 to 5%, following AS/SP plus or minus 14 days of primaquine (26), a sample size of 40 patients would be needed in each treatment arm.

Data were entered using EpiData (version 3.1,EpiData Software, Denmark) and analysed using Stata version 14 (Stata Corp., USA). The primary analysis was an intention to treat (ITT) analyses including all participants enrolled into the study. A secondary analysis was done using a modified intention to treat (mITT) approach in which patients who had received sub-optimal treatment dosage were excluded. Sub-optimal drug dosage was defined as a total dose below 4 mg/kg for AS, below 25 mg/kg for Sulphadoxine and 1.25 mg/kg for pyrimethamine [13, 14]. The sub-optimal PQ dose was defined as below 0.15 mg/kg in the Pf-PQ1 arm and below 2.5 mg/kg total dose of primaquine in the Pv-PQ14 arm [25].

Normally distributed data were compared using Student’s t-test, the t-test for paired samples or one-way analysis of variance, and non-parametric comparisons were made using the Mann–Whitney U test or Wilcoxon signed rank test for paired samples. Proportions were examined using χ^2^ with Yates’ correction or Fisher’s exact test. Correlations were assessed using the Pearson test for correlated proportions for normal distributed variables and the Spearman rank test for non-normal distributed variables. Efficacy endpoints were assessed by survival analysis, in which the cumulative risk of failure was calculated by the Kaplan–Meier product limit formula [26]. The equality of survivor functions was tested using the log-rank test (exponential scores test).

The results of the survival analysis were adjusted according to parasite genotyping results of the admission and recurrent parasitaemia. Patients with recurrent parasitaemia due to a different species identified on admission or a PCR confirmed re-infection were censored at the day of occurrence. Following a conservative approach indeterminate PCR results were considered treatment failures. Parasite clearance was presented as the proportion of patients with microscopy negative results within the first 3 days, missing data were treated as suggested by the World Wide Antimalarial Resistance Network (WWARN) [27].

Safety analysis included the risks of a drop in Hb concentration greater than 25%, severe anaemia (Hb < 7 g/dl), requiring a blood transfusion, and the proportion of patients with adverse and serious adverse events. Haematological response was assessed from the fractional fall in Hb between baseline and days 2, 7 and 16. The effect of malaria species, peripheral parasitaemia, G6PD activity and total dose of primaquine on the degree of haemolysis was assessed by multivariable linear regression using a backward selection of variables [28]. The performance of the Biosensor was assessed using standard formulae [29, 30] in which the adjusted male median (AMM) was considered 100% G6PD activity [15].

## Results

### Study profile and baseline characteristics

**Fig. 1:**
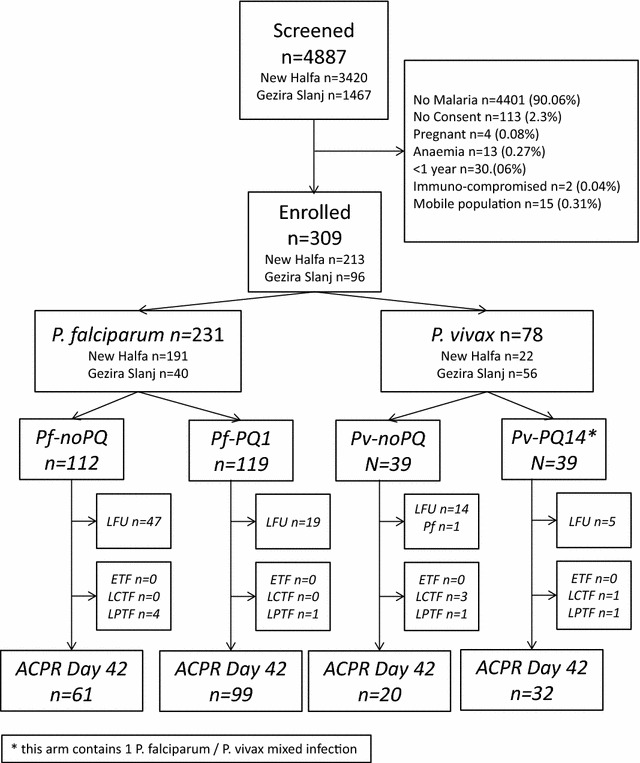
Study profile. *ETF* early treatment failure, *LCTF* late clinical treatment failure, *LPTF* late parasitological treatment failure, *ACPR* adequate clinical and parasitological response

Between 7th December 2015 and 9th March 2016, 4887 patients were screened for malaria. Overall 486 (9.9%) patients had malaria, of whom 309 (63.6%) were enrolled in the study (213 [69.9%] in New Halfa and 96 [31.1%] in Gezira Slanj) (Fig. 1). The main reason for non-enrolment of patients with malaria, was declining to participate in the study (113/177, 63.8%). Other reasons are listed in Fig. 1. No patient was excluded from 14 day primaquine treatment because of low G6PD activity as measured by spectrophotometry.

Based on microscopy a total of 231 (74.8%) patients were infected with *P. falciparum,* 77 (24.9%) with *P. vivax* and one patient (0.3%) with a mixed infection of *P. falciparum* and *P. vivax*. Patients with *P. falciparum* infections had a significantly higher parasitaemia and were less likely to be gametocytaemic at enrolment than those with *P. vivax* infection (Table 1). Baseline characteristics between the randomized groups for each species were similar, proportions of patient enrolled into each arm were similar per site (Table 1).

**Table 1:** Baseline characteristics

	*P. falciparum*				*P. vivax* ^a^			
	All N = 231	Pf-noPQ N = 112	Pf-PQ1 N = 119	p	All N = 78	Pv-PQ14 N = 39	Pv-noPQ N = 39	p
N (%) male	117 (50.6%)	53 (47.3%)	65 (53.7%)	0.329	40 (51.3%)	20 (51.3%)	20 (51.3%)	1.000
New Halfa (%)	191 (100.0)	92 (48.2)	99 (51.8)	0.833	22 (100.0)	12 (54.5)	10 (45.5)	0.615
Gezira Slanj (%)	40 (100.0)	20 (50.0)	20 (50.0)		56 (100.0)	27 (48.2)	29 (51.8)	
Median age in years, (IQR)	19.0 (12.0–28.0)	18.5 (12.0–28.0)	19.0 (12.0–28.0)	0.310	17.0 (10.0–27.0)	16.0 (10.0–23.0)	19.0 (9.0–30.0)	0.272
0–5 years, N (%)	18 (7.8%)	8 (44.4%)	10 (55.5%)	0.824	4 (5.1%)	3 (75.0%)	1 (25%)	0.615
> 5 years, N (%)	213 (92.2%)	104 (48.8%)	109 (51.2%)		74 (94.1%)	36 (48.6%)	38 (51.4%)	
Body Temp. mean °C (95% CI)	38.8 (38.7–38.8)	38.8 (38.7–38.9)	38.8 (38.8–38.9)	0.499	38.6 (38.4–38.7)	38.5 (38.4–38.7)	38.5 (38.4–38.7)	0.415
Median Hb in g/dl (IQR)	11.7 (10.8–12.8)	11.7 (10.7–12.8)	11.8 (10.9–12.8)	0.535	11.0 (10.4–13.0)	12.0 (10.9–13.0)	11.0 (10.0–12.3)	0.113
Anaemia (< 10 g/dl), N (%)	13 (5.6%)	8 (7.1%)	5 (4.2%)	0.332	8 (10.3%)	4 (10.3%)	4 (10.3%)	1.000
Weight, mean kg (95% CI)	57.4 (54.0–60.7)	56.7 (51.8–61.6)	58.0 (53.2–62.7)	0.357	47.2 (42.4–52.1)	44.3 (38.3–50.3)	50.1 (42.3–58.0)	0.119
Parasitaemia (µl^−1^), geometric mean (95% CI)	7840 (7443–8259)	7779 (7226–8375)	7898 (7333–8508)	0.321	6939 (6383–7544)	6933 (6164–7797)	6947 6135–7865)	0.956
Gametocytaemia (µl^−1^), geometric mean (95% CI)	0	0	0	1.000	252 (224–284)	245 (203–296)	259 (222–303)	0.449
Gametocytaemia, N (%)s	0 (0.0%)	0 (0.0%)	0 (0.0%)	1.000	78 (100.0%)	39 (100.0%)	39 (100.0%)	1.000
Mean G6PD activity (U/gHb) (95% CI)	9.95 (9.52–10.38)	10.06 (9.37–10.74)	9.85 (9.30–10.39)	0.316	9.57 (8.27–10.88)	9.79 (7.85–11.73)	9.40 (7.35–11.44)	0.381

^a^Includes one patient with *P. falciparum/P. vivax* mixed infection

The target dose of AS was achieved in 100.0% patients, but only 59.5% (184/309) of patients received the target dose of S/P (Table 2). The median dose of PQ prescribed for patients in the Pf-PQ1 arm was 0.25 mg/kg (IQR 0.192–0.333) with 5 patients (4.2%) receiving less than 0.15 mg/kg. The median total dose of PQ prescribed in the Pv-PQ14 arm was 4.20 mg/kg (IQR 3.18–4.67), with 15.4% (6/39) patients prescribed a total dose less than 2.5 mg/kg [26]. Pill counts were undertaken in all patients receiving 14 days unsupervised PQ (Pv-PQ14) at the end of their treatment course. One participant (2.6%) had only taken 2 of the 14 doses of PQ, but no pills were remaining for any of the other patients.

**Table 2:** Treatment provided (in mg/kg bodyweight)

	Artesunate	Sulphadoxine	Pyrimethamine	Primaquine^b^
Target dose [13]	4 mg/kg per day for 3 days (range 2–10 mg/kg)	25 mg/kg single dose (range 25–70 mg/kg)	1.25 mg/kg single dose (range 1.25–3.5 mg/kg)	0.25 mg/kg or 3.5 total dose
*P. falciparum* (n = 231)				
Mean total dose received in mg/kg (95% CI)	10.3 (9.77–10.74)	34.26 (31.37–37.15)	1.7 (1.57–1.86)	0.28 (0.254–0.305)
Median total dose received in mg/kg (IQR, range)	9. 23 (7.69–12.00, 5.36–25.00)	25.42 (19.23–41.67, 13.39–115.38)	1.3 (0.96–2.08, 0.67–5.77)	0.25 (0.192–0.333, 0.000–0.789)
N (%) patients with suboptimal dosage^a^	0 (0.0%)	104 (45.0%)	104 (45.0%)	5 (4.2)
*P. vivax/mixed infection* (n = 78)				
Mean total dose received in mg/kg (95% CI)	10.9 (10.32–11.64)	39.3 (34.76–43.89)	2.0 (1.74–2.2.)	4.2 (3.57–4.86)
Median total dose received in mg/kg (IQR, range)	10.81 (9.23–7.06, 6.12–27.78)	30.0 (23.07– 55.56, 15.31–111.11)	1.5 (1/15–2.78, 0.77–5.56)	4.2 (3.18–4.67, 0.12–11.67)
N (%) patients with suboptimal dosage^a^	0 (0.0%)	21 (26.9)	21 (26.9)	6 (15.4%)

^a^Suboptimal dosage was defined as a total dose below 4 mg/kg AS, below 25 mg/kg sulphadoxine, below 1.25 mg/kg pyrimethamine and below 0.15 mg/kg (Pf) or 2.5 mg/kg (Pv) primaquine

^b^Only patients from Pf-PQ arm (n = 119, 0.25 mg/kgBW) and Pv-PQ14 (n = 39, 3.5 mg/kgBW) arm included

### Early therapeutic response

Of the 231 patients with *P. falciparum* infection, 74.7% (130/174) were still parasitaemic on day 1, and this fell to 4.6% (8/174) on day 2. One 10-year old patient with a baseline parasitaemia of 2750 parasites/µl was afebrile, but still parasitaemic (475 µl^−1^) on day 3; he was subsequently lost to follow up. Fever clearance in all other patients with *P. falciparum* infection was rapid, with 95.4% (124/130) patients being afebrile by day 2.

Parasite clearance in patients with *P. vivax* was slower than that for *P. falciparum.* Of the 78 patients with *P. vivax* or mixed infection, 96.8% (60/62) were still parasitaemic on day 1 and 17.7% (11/62) on day 2. One 35-year old patient (1.6%) with a baseline parasitaemia of 9300 parasites/µl was afebrile, but remained parasitaemic (645 µl^−1^) on day 3; the patient was subsequently lost to follow up. All patients with *P. vivax* parasitaemia had gametocytes present at baseline, but by day 2 this proportion had fallen to 1.6% (1/62). Fever clearance was fast with only one patient with *P. vivax* being febrile by day 2.

### Efficacy outcomes

A total of 85 (27.5%) patients were lost to follow up before the end of the study and one patient (0.32%) presented with *P. falciparum* infection on day 28 after being enrolled with a *P. vivax* infection (Fig. 1). There were no early treatment failures in any treatment arm, but 11 (3.6%) patients represented with late treatment failure (LTF), 7 of which were classified as LPTF and 4 as LCTF (Fig. 1).

#### *Plasmodium falciparum* arms

In the *P. falciparum* arm, a total of 5 patients had recurrent parasitaemia on days 7 (n = 2), 14 (n = 2) and 35 (n = 1). The overall risk of recurrent parasitemia on day 28 was 2.0% [95% CI 0.75–5.19%], the risk being greater in patients treated with Pf-noPQ (3.4% [95% CI 1.1–10.3%]) compared to 0.9% (95% CI 0.1–6.0%) in those treated with Pf-PQ1; Hazard Ratio HR = 0.26 [95% CI 0.03–2.47], p = 0.203.

**Fig. 2:**
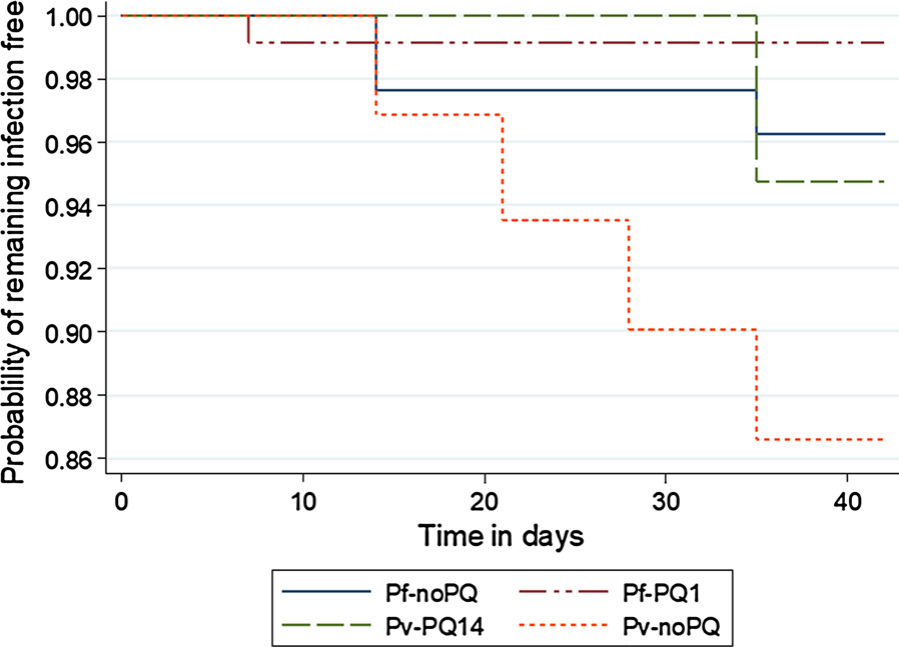
Cumulative risk of patients with recurrent parasitaemia (PCR corrected)

By day 42 the risk of parasite recurrence was 2.5% [95% CI 2.06–6.01%]; 4.8% (95% CI 1.8–12.4%) in the Pf-noPQ and 0.9% (95% CI 0.1–6.0%) in the Pf-PQ14 (HR = 0.19 [95 CI 0.02–1.67], p = 0.092); Fig. 2. PCR demonstrated one patient with a reinfection in the Pf-noPQ arm, with the 4 other recurrent samples of the Pf-PQ1 arm providing indeterminate results. The PCR adjusted risk of failure on day 28 was 2.4% (95% CI 0.6–0.9%) and 3.8% (1.2–11.2%) on day 42 (HR = 0.25 [95 CI 0.03–2.38], p = 0.188) for the Pf-noPQ arm. Site specific efficacy estimates are summarized in Table 3, efficacy between sites for the individual arms did not vary significantly (all p > 0.05). No patients initially infected with *P. falciparum* had gametocytes detected on blood film examination during follow up.

**Table 3:** Failure rates overall and per site on day 28 and 42 using the ITT and mITT populations

	*P. falciparum (PCR corrected)*				*P. vivax*			
	Pf-noPQ	Pf-PQ1	p value	Hazards ratio	Pv-noPQ	Pv-PQ14	p value	Hazards ratio
ITT								
D28								
Overall (95% CI)	2.35 (0.59–9.08)	0.86 (0.12–5.96)	0.419	0.385 (0.035–4.241)	9.93 (3.31–27.76)	0.00	*0.046*	–
New Halfa (95% CI)	2.90 (0.73–11.10)	1.03 (0.15–7.09)	0.406	0.374 (0.034–4.129)	20.0 (3.08–79.62)	0.00	0.157	_
Gezira Slanj (95% CI)	0.00	0.00	1.000	–	8.17 (2.11–28.92)	0.00	0.120	_
p value^b^	0.493	0.658	–	–	0.487	1.000	–	–
D42								
Overall (95% CI)	3.75 (1.22–11.22)	0.86 (0.12–5.96)	0.189	0.247 (0.026–2.378)	13.39 (5.24–31.91)	5.26 (1.34–19.44)	0.212	0.358 (0.066–1.956)
New Halfa (95% CI	4.66 (1.52–13.85)	1.03 (0.15–7.09)	0.176	0.238 (0.025–2.293)	20.0 (3.08–79.62)	0.00	0.157	–
Gezira Slanj (95% CI)	0.00	0.00	1.000	–	12.35 (4.15–33.59)	7.14 (1.84–25.65)	0.480	0.535 (0.089–3.200)
p value^b^	0.389	0.658	–	–	0.392	0.684	–	–
mITT^a^								
D28								
Overall (95% CI)	2.00 (0.28–13.36)	0.00	0.282	–	0.00	0.00	1.000	–
New Halfa (95% CI)	2.86 (0.41–18.60)	0.00	0.237	–	0.00	0.00	1.000	–
Gezira Slanj (95% CI)	0.00	0.00	1.000	–	0.00	0.00	1.000	–
p value^b^	0.513	1.000	–	–	1.000	1.000	–	–
D42								
Overall (95% CI)	4.28 (1.08–16.10)	0.00	0.117	–	0.00	6.90 (1.77–24.86)	0.247	–
New Halfa (95% CI)	6.21 (1.58–22.73)	0.00	0.083	–	0.00	0.00	1.000	–
Gezira Slanj (95% CI)	0.00	0.00	1.000	–	0.00	10.00 (2.60–34.40)	0.186	–
p value^b^	0.340	1.000	–	–	1.00	0.334	0.334	–

Significant values are in italics (p < 0.05)

^a^mITT population excluded 132 (42.7%) with incomplete or suboptimal dosing of study drugs

^b^This p value refers to site specific differences and does not consider overall results

#### *Plasmodium vivax* arms

In the *P. vivax* arms, a total of 6 patients had recurrent parasitaemia at days 14 (n = 1), 21 (n = 1), 28 (n = 1), 35 (n = 3). In patients not receiving 14 days primaquine the risk of recurrent *P. vivax* at day 28 was 9.9% [95% CI 3.3–27.8%] compared to 0% in those treated with AS/SP plus 14 days primaquine; p = 0.046. At day 42 the respective risks had risen to 13.4% [95% CI 5.2–31.9%] in the Pv-noPQ arm and 5.3% [95% CI 1.3–19.4%] in the Pv-PQ14 arm; HR 0.36, [95% CI 0.1–2.0], p = 0.212 (Fig. 2). There was no difference in treatment efficacy between sites (Table 3).

### Modified intention to treat analysis and optimal versus suboptimal treatment

In the mITT analysis, 41.7% (129/309) of patients receiving a sub-optimal dose of any of the study drugs were excluded, leaving 127 (54.9%) of patients with *P. falciparum* and 53 (67.9%) patients with *P. vivax* (Table 3). The overall risk of recurrent parasitaemia was lower in patients who had received optimal treatment, although this did not reach statistical significance in any arm by day 42.

No patient received sub-optimal AS (Table 2). For patients of the Pv-noPQ arm who received less than 25 mg/kg SP, the risk of recurrence was 39.4% [95% CI 16.90–74.20] compared to 0.0% in patients of the same arm who received higher treatment doses (p = 0.003). No significant differences in efficacy were found when comparing sub-optimal versus optimal SP treatment in the other treatment arms.

### Haematological response

**Fig. 3:**
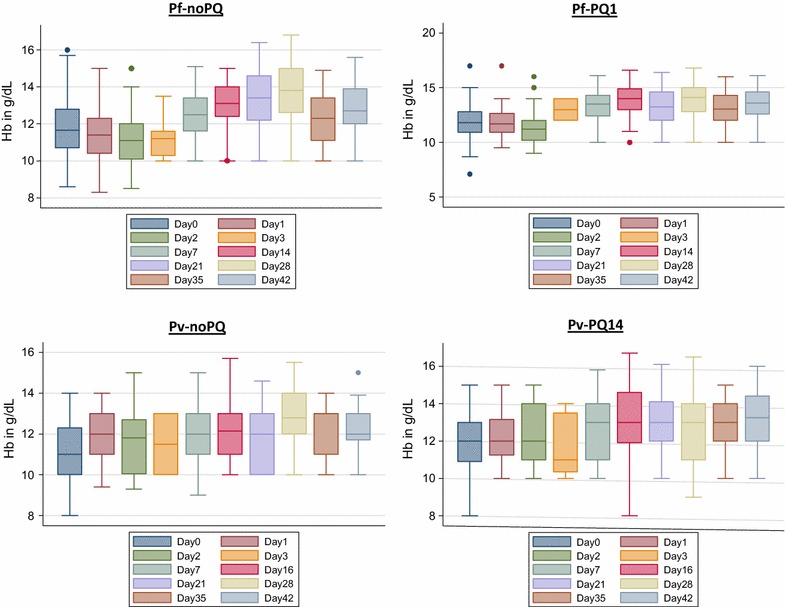
Fractional change of Hb between baseline and day of follow up

At baseline the median Hb concentration was 11.7 g/dl in patients with *P. falciparum* and 11.0 g/dl in patients with *P. vivax* (Table 1). The greatest median fractional fall in Hb occurred in the *P. falciparum* arms between baseline and day 2 prior to administration of any primaquine, whereas there was no median drop in Hb among patients with a *P. vivax* infection (Table 4). On day 2 patients infected with *P. falciparum* had a median fractional change in Hb of − 4.9% (IQR − 10.6 to + 0.9, range − 29.3 to + 50.7), compared to patients infected with *P. vivax* whose Hb did not change (IQR − 7.1 to + 7.7 to 4.6, range − 23.1 to + 50.0) over the same period; p = 0.015 (Fig. 3). A total of 1.7% (4/231) of patients with *P. falciparum* had a fractional fall in Hb greater than 25% between day of enrolment and day 2, while this was not the case for any patient with *P. vivax* infection (p = 0.316).

**Table 4:** Relative change in the mean Hb concentration between baseline and day of follow up

Relative median change in Hb from baseline	All	*P. falciparum*			All	*P. vivax*		
		Pf-noPQ	Pf-PQ1	p		Pv-noPQ	Pv-PQ14	p
D1 (IQR, n)	− 2.7% (− 9.09 to 1.92, 174)	− 3.8% (− 7.75 to 2.27, 86)	− 2.42% (− 9.33 to 0.00, 88)	0.8161	0.0% (− 5.30 to 0.92, 62)	0.0% (− 4.72 to 0.00, 30)	0.0% (− 5.68 to 8.71, 30	0.219
D2 (IQR, n)	− 4.9% (− 10.62 to − 0.87, 163)	− 4.4% (− 9.05 to 0.43, 72)	− 5.6% (− 11.9 to 2.65, 91)	0.525	0.0% (− 7.14 to 7.69, 61)	0.0% (− 7.14 to 1.63, 32)	0.0% (− 7.69 to 9.09, 29)	0.460
D7 (IQR, n)	11.2% (0.00 to 19.54, 164)	6.0% (− 0.76 to 14.81, 62)	13.2% (0.00 to 23.89, 102)	*0.007*	8.0% (0.00 to 15.38, 56)	0.0% (0.00 to 10.00, 25)	9.1% (0.00 to 15.38, 31)	0.106
D14 (IQR, n)	16.5% (4.27 to 27.08, 162)	13.3% (0.00 to 22.69, 57)	18.6% (7.69 to 30.08, 105)	*0.006*	NA	9.1% (NA, 1)	n = 0	NA
D16 (IQR, n)	n = 0	n = 0	n = 0	NA	7.7% (0.00 to 18.18, 67)	7.7% (0.00 to 18.18, 30)	7.7% (0.00 to 18.18, 37)	1.000
D21 (IQR, n)	15.4% (0.85 to 23.08, 147)	15.0% (0.00 to 26.85, 61)	15.4% (4.07 to 22.76, 86)	0.939	8.3% (0.00 to 18.18, 55)	7.7% (0.00 to 18.18, 23)	8.7% (0.00 to 18.18, 32)	0.614
D28 (IQR, n)	16.9% (4.41 to 26.46, 140)	13.5% (0.83 to 24.77, 62)	17.7% (6.40 to 28.46, 78)	0.284	11.1% (0.00 to 21.10, 47)	10.0% (0.00 to 18.18, 21)	12.5% (0.00 to 21.10, 26)	0.754

Significant values are in italics (p < 0.05)

The median Hb levels increased in all treatment arms between days 0 and 7 (Fig. 3, Table 4). In patients with *P. falciparum* the Hb concentration at day 7 had risen 6.0% (IQR − 0.8 to 14.8) from baseline following treatment with Pf-noPQ compared to 13.2% (IQR 0.0 to 23.9) after treatment with Pf-PQ1; p = 0.007. In patients with *P. vivax*, there was no change in Hb (IQR 0.0 to 10.0) between day 0 and day 7 following treatment with Pv-noPQ compared to 9.1% (IQR 0.0 to 15.4) in vivax patients treated with primaquine (Pv-PQ14); p = 0.106. None of the patients included required a blood transfusion.

### Tolerability

A total of 551 adverse events (AE) were reported, of which 497 (90.2%) were categorized as mild and 51 (9.8%) as moderate. Three (0.5%) mild events were recorded as possibly related to AS/SP. The most frequent AEs were gastrointestinal symptoms including poor appetite (40.3%), nausea (25.6%) and abdominal pain (20.7%) (Table 5). In 93.8% (226/241) of cases the first episode of gastrointestinal symptoms was reported on or after day 2. In total 68.9% (82/119) of patients treated with Pf-PQ1 reported at least one gastrointestinal AE on or after day 2 compared to 81.3% (91/112) of those with Pf-noPQ; relative risk = 0.820 [95% CI 0.631–1.065], p = 0.155. The corresponding relative risk in patients from the Pv-PQ14 arm compared to patients from the Pv-noPQ arm was 0.943 [95% CI 0.592–1.50], p = 0.808. No serious adverse events occurred in any study patient.

**Table 5:** Overview of main adverse events

Type of AE	Total	*P. falciparum*			*P. vivax*		
		Pf-noPQ	Pf-PQ1	p	Pv-noPQ	Pv-PQ14	p
Poor appetite, n (%)	222 (40.3)	93 (47.2)	75 (35.7)	*0.012*	30 (41.1)	24 (33.8)	0.366
Nausea, n (%)	145 (25.6)	58 (29.4)	60 (28.6)	0.533	13 (17.8)	14 (19.7)	0.769
Abdominal pain, n (%)	114 (20.7)	29 (14.7)	51 (24.3)	*< 0.001*	21 (28.8)	13 (18.3)	0.140
Vomiting, n (%)	37 (6.7)	15 (7.6)	13 (6.2)	0.536	5 (6.8)	4 (5.6)	0.518
Rash, n (%)	1 (0.2)	0 (0.0)	1 (0.5)	0.521	0 (0.0)	0 (0.0)	1.000
Other, n (%)	36 (6.5)	2 (1.0)	14 (6.7)	*0.004*	4 (5.5)	16 (22.5)	*0.004*
Total, n (%)	551	197	210		73	71	

Significant values are in italics (p < 0.05)

### G6PD activity and performance of the biosensor (AccessBio–CareStart)

**Fig. 4:**
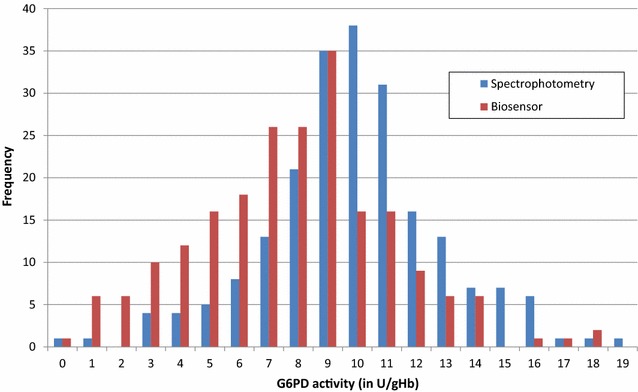
G6PD activity distribution within the study population/assay. All values are rounded to the nearest integer

G6PD activity was quantified by spectrophotometry and Biosensor™ in 68.5% (213/309) patients. The adjusted male median by spectrophotometry was 9.7 U/gHb, compared to 7.9 U/gHb derived by the Biosensor™ (p < 0.001). By spectrophotometry 2 (0.9%) patients with *P. falciparum* infection had G6PD activities less than 10% of the AMM, no patient had G6PD activities between 10 and 30% and 18 (8.5%) patients had G6PD activity between 30 and 60% (Fig. 4).

**Fig. 5:**
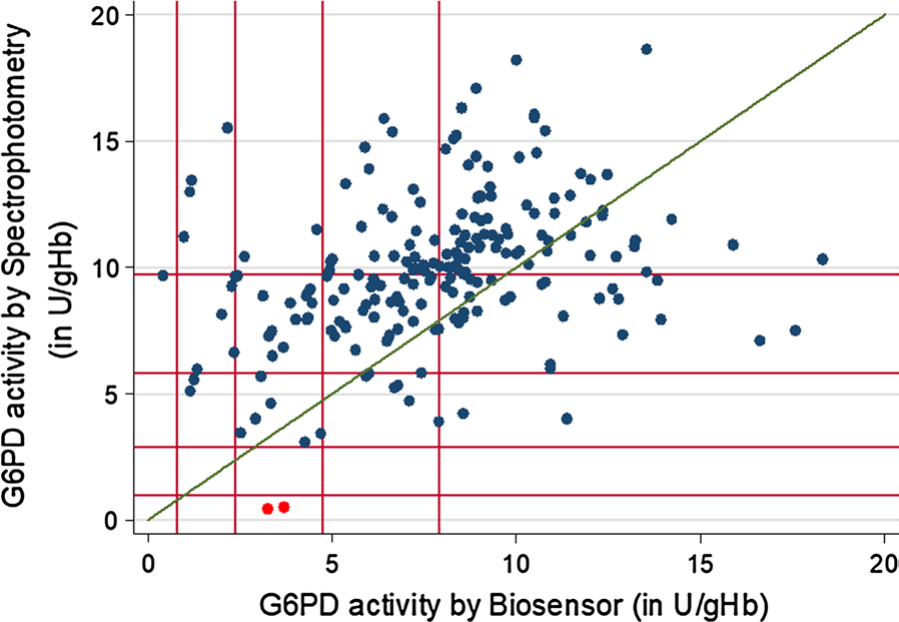
Scatter plot biosensor result versus spectrophotometry. Green line = line of equality, red lines from origin outwards represent 10, 30, 60 and 100% G6PD activity/assay, red markers = subjects with < 30% activity, not recognized by the Biosensor

The Biosensor™ identified 1 patient with less than 10% G6PD activity, 11 participants (5.2%) with activities between 10 and 30% activity, and 27 patients with G6PD activity between 30 and 60% (Table 6, Fig. 5). None of the patients with G6PD activities below 10% were identified by the biosensor correctly. The correlation between the activity recorded by the two assays was r_s_ = 0.330 (p < 0.001), with a mean difference of 2.1 U/gHb and the 95% limit of agreement (LoA) ranged from − 5.1 U/gHb to 9.2 U/gHb (Figs. 4, 5). In the absence of true G6PD deficient results with activities < 30% of the AMM sensitivity could not be calculated, while specificity was 0.94 (95% CI 0.90–0.97). Sensitivity and specificity at 60% of the AMM were 0.55 (95% CI 0.32–0.77) and 0.86 (95% CI 0.80–0.91), respectively.

**Table 6:** Categorized G6PD result/assay

G6PD cut off	Biosensor™				Total
	< 10%	10 to < 30%	30 to < 60%	≥ 60%	
Spectrophotometry					
< 10 %	0	0	2	0	2
10 to < 30 %	0	0	0	0	0
30 to < 60 %	0	2	7	9	18
≥ 60 %	1	9	17	166	193
Total	1	11	27	176	213

## Discussion

This efficacy study demonstrates that when the target dose of AS/SP was achieved the risk of recurrent parasitaemia was low for patients with either *P. falciparum* or *P. vivax* in the two Sudanese study sites assessed. The dosage scheme applied followed recommendations of the WHO; never the less almost half of the patients received a dose of SP less than the intended target dose of 25 mg/kg [13]. While the target dose is designed for patients with a maximum body weight of 60 kg, a substantial fraction of the study population weighed more, resulting in under dosing (Additional file 1) and in patients with *P. vivax* treated with AS/SP alone, this was associated with an almost 40% risk of recurrence by day 42. The additional administration of single dose primaquine in patients with *P. falciparum* and 14 days primaquine in *P. vivax*, was associated with lower risks of parasite recurrence (5.3- and 2.8-fold, respectively), although these benefits did not reach statistical significance. Results on gametocyte carriage are limited by the small denominator applied for microscopy (200WBC).

In 2006, a study in the east of the country reported a day 28 PCR corrected efficacy of 93.5% for AS/SP for *P. falciparum* [31]. Other clinical studies conducted between 2010 and 2015 at six sites suggest that the PCR corrected efficacy of AS/SP against *P. falciparum* by day 28 generally exceeds 95%, except for one site in Gedaref, in the East of the country, where the efficacy fell from 91% in 2012 to 87% in 2015 [32].

One study from Kassala, Eastern Sudan reported a PCR corrected efficacy of 93.7% by day 28 [33], while a study from Damazin, South-Eastern Sudan reported a PCR corrected efficacy of 80% for AS/SP by day 28 [34]. The study in Kassala also identified a high proportion of parasites with double mutations in the *pfdhfr* and *pfdhps* genes and a single case with a triple mutation in the *pfdhfr* gene, all associated with antifolate resistance [33].

In Somalia, the efficacy of AS/SP has fallen to 88% and artemether–lumefantrine has replaced AS/SP as the first-line treatment for *P. falciparum* [35]. In March 2017, after patient enrolment to the current study had been completed, North Sudan anti-malarial policy was also changed from AS/SP to artemether–lumefantrine (AL) for the treatment of uncomplicated malaria.

In areas co-endemic for *P. falciparum* and *P. vivax* AS/SP has been used as a universal schizontocidal treatment for both species particularly in locations where diagnostic facilities are unable to distinguish reliably between species. To date 3 published trials have assessed the efficacy of AS/SP against *P. vivax*. Tjitra et al. found AS/SP to have almost 90% efficacy against *P. vivax* in Papua, Indonesia [36]. However clinical trials with longer follow up revealed that the risk of recurrent *P. vivax* by day 42 after AS/SP rose to 25% in Afghanistan [37], and 67% in Papua New Guinea [38]. In the current study, the overall risk of *P. vivax* recurrence at day 42 was 13.4% in patients treated with AS/SP alone, less than half that expected in our a priori power calculation. The risk of recurrence fell to 5.3% in those treated with AS/SP plus 14 days primaquine although this did not reach statistical significance (p = 0.212); Table 3. A longer follow-up and larger sample size would be required to document the additional benefits of primaquine radical cure that appeared to be emerging [39].

WHO guidelines recommend that the radical cure of *P. vivax* can be achieved with a 14-day regimen of primaquine, although the long course of treatment is associated with poor adherence and compromised effectiveness [25, 40–44]. In some settings unsupervised PQ appears to work well [45], and indeed in the current study pill counting, undertaken on day 16, suggested excellent adherence to the 14-day treatment regimen, with only one patient out of 39 failing to take all of their tablets. However, the nature of a clinical trial including regular review may influence patients behaviour significantly, leading to an overestimation of the true clinical effectiveness in non-trial settings.

PQ is known to cause haemolysis in G6PD deficient individuals with the nadir usually observed 2–7 days after initial exposure [46]. The greatest fall in Hb was observed at day 2 prior to the administration of any primaquine and thus is likely to be attributable to parasite rather than drug induced haemolysis. In patients with *P. falciparum* infection there was no further fall in Hb observed between day 2 and day 7, with or without a single dose of primaquine. These observations are reassuring and concur with previous reports from Tanzania [47] supporting the current WHO guidelines that do not recommend routine G6PD testing prior to the administration of a single dose of PQ [13]. None of the patients with *P. vivax* infection had G6PD activity below 30%, although 2/22 (9.1%) had intermediate activity between 30 and 60%, reassuringly none of the patients had a significant fall in Hb after day 2.

Howes et al. had earlier documented a country wide G6PD deficiency prevalence of 15.3% with higher frequencies towards the West of the country and lower frequencies in the East [48]. These prevalence estimates are based on 8 surveys and a definition of G6PD deficiency using a cut-off of 30% [15]. G6PD enzyme activity was only assessed in patients enrolled in New Halfa in the East of the country and defined following the same cut-off. Based on this definition only two patients (0.9%) were G6PD deficient. This lower prevalence is partly explained by the location of sample collection which was close to the borders with Eritrea and Ethiopia, where the prevalence of G6PD deficiency is low [48]. Furthermore, it is likely that G6PD deficiency offers some degree of protection against clinical malaria [49–54], and the patients with deficiency identified in cross sectional surveys may be less likely to present to clinics with symptomatic illness.

The study also evaluated a novel point of care Biosenor™ (Accessbio/Carestart, USA) which was compared to the reference method of spectrophotometry. The results highlight that the current Biosensor™ underdiagnosed severe G6PD deficiency, a finding consistent with a recent similar study conducted in Bangladesh [21]. Out of the 21 participants with G6PD activities below 60% by spectrophotometry only 11 (52.4%) were correctly classified with both patients with severe G6PDd classified as intermediate deficiency using the Biosensor™.

## Conclusion

AS/SP appears to retain adequate efficacy in uncomplicated malaria in New Halfa and Gezira Slanj. Both a single dose of primaquine in patients with *P. falciparum* and a 14-day regimen in patients with *P. vivax* were well tolerated, and associated with a modest reduction in the number of recurrences by day 42, however reduction was not significant for *P. falciparum*. The Biosensor™ achieved suitable operational characteristics, but test performance is insufficient for routine diagnosis. A second generation with integrated haemoglobin measurement will be introduced to the market and may provide a more suitable solution.

## Additional files

**Additional file 1.** Scatter plot body weight vs. total dose SP received. red line = target dose recommended by the WHO.

**Additional file 2.** Database.
