# Supplementary figures and images for: Low risk of recurrence following artesunate–Sulphadoxine–pyrimethamine plus primaquine for uncomplicated Plasmodium falciparum and Plasmodium vivax infections in the Republic of the Sudan
Source: Malar J. 2018 Mar 16;17:117. doi: 10.1186/s12936-018-2266-9 (PMC5857106; doi:10.1186/s12936-018-2266-9)

## Slide 1
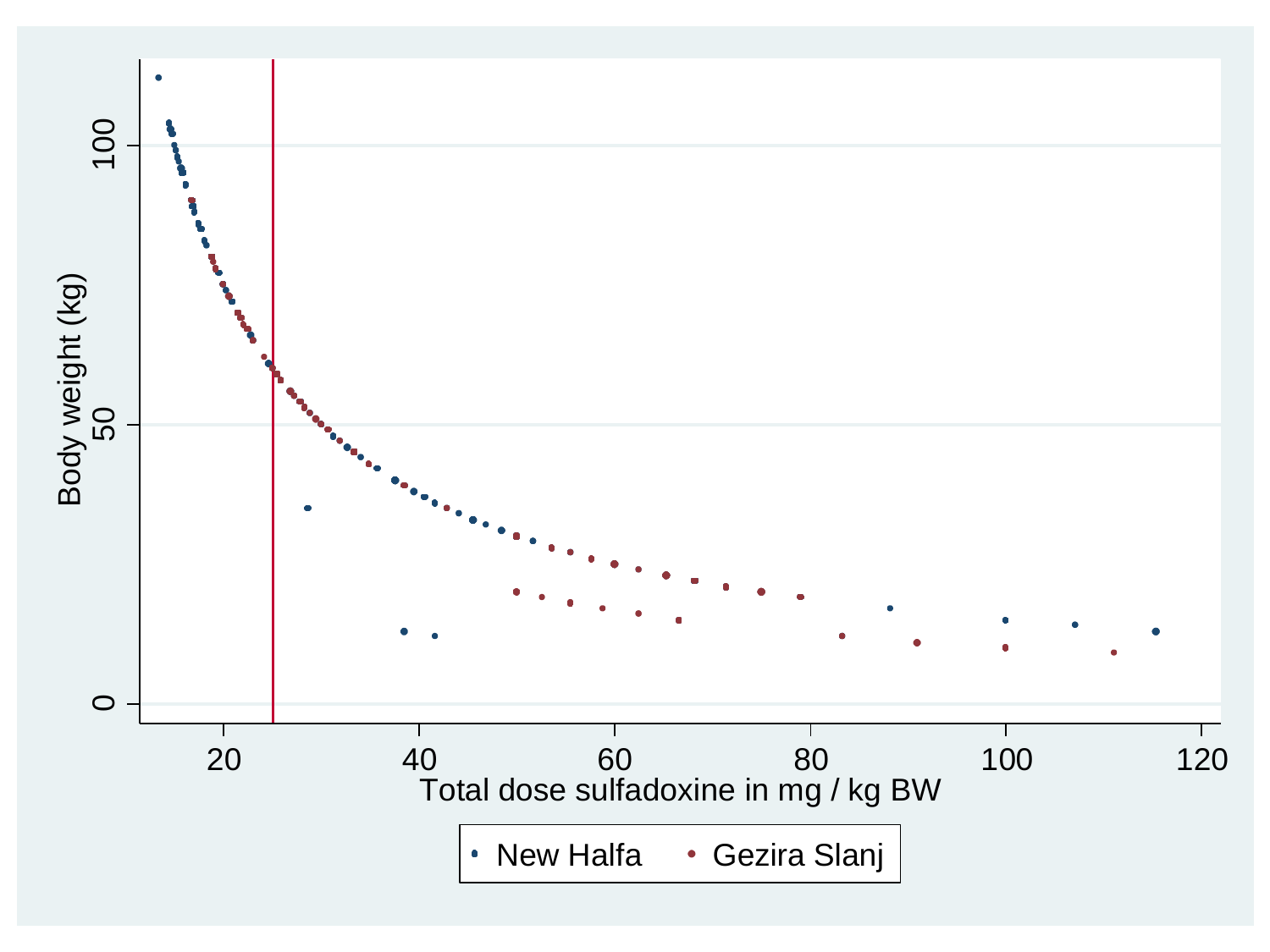

Supplement: Supplementary file 1 — Additional file 1. Scatter plot body weight vs. total dose SP received. red line = target dose recommended by the WHO. [file 12936_2018_2266_MOESM1_ESM.pptx]
